# Design of Targeted B Cell Killing Agents

**DOI:** 10.1371/journal.pone.0020991

**Published:** 2011-06-06

**Authors:** Alexey V. Stepanov, Alexey A. Belogurov, Natalia A. Ponomarenko, Oleg A. Stremovskiy, Leonid V. Kozlov, Anna M. Bichucher, Sergey E. Dmitriev, Ivan V. Smirnov, Olga G. Shamborant, Dmitry S. Balabashin, Lidia P. Sashchenko, Alexander G. Tonevitsky, Alain Friboulet, Alexander G. Gabibov, Sergey M. Deyev

**Affiliations:** 1 M.M. Shemyakin and Yu.A. Ovchinnikov Institute of Bioorganic Chemistry, Russian Academy of Sciences, Moscow, Russia; 2 Institute of Gene Biology, Russian Academy of Sciences, Moscow, Russia; 3 G.N. Gabrichevsky Research Institute of Epidemiology and Microbiology, Moscow, Russia; 4 Belozersky Institute of Physico-Chemical Biology, Moscow State University, Moscow, Russia; 5 Faculty of Biology, Lomonosov Moscow State University, Moscow, Russia; 6 Université de Technologie de Compiègne, Compiègne, France; Institute of Microbial Technology, India

## Abstract

B cells play an important role in the pathogenesis of both systemic and organ-specific autoimmune diseases. Autoreactive B cells not only produce autoantibodies, but also are capable to efficiently present specific autoantigens to T cells. Furthermore, B cells can secrete proinflammatory cytokines and amplify the vicious process of self-destruction. B cell-directed therapy is a potentially important approach for treatment of various autoimmune diseases. The depletion of B cells by anti-CD20/19 monoclonal antibody Retuximab® used in autoimmune diseases therapy leads to systemic side effects and should be significantly improved. In this study we designed a repertoire of genetically engineered B cell killers that specifically affected one kind of cells carrying a respective B cell receptor. We constructed immunotoxins (ITs), fused with c-myc epitope as a model targeting sequence, based on barnase, *Pseudomonas* toxin, Shiga-like toxin *E.coli* and Fc domain of human antibody IgGγ1. C-MYC hybridoma cell line producing anti-c-myc IgG was chosen as a model for targeted cell depletion. C-myc sequence fused with toxins provided addressed delivery of the toxic agent to the target cells. We demonstrated functional activity of designed ITs in vitro and showed recognition of the fusion molecules by antibodies produced by targeted hybridoma. To study specificity of the proposed B cells killing molecules, we tested a set of created ITs ex vivo, using C-MYC and irrelevant hybridoma cell lines. *Pseudomonas*-containing IT showed one of the highest cytotoxic effects on the model cells, however, possessed promiscuous specificity. Shiga-like toxin construct demonstrated mild both cytotoxicity and specificity. Barnase and Fc-containing ITs revealed excellent balance between their legibility and toxic properties. Moreover, barnase and Fc molecules fused with c-myc epitope were able to selectively deplete c-myc-specific B cells and decrease production of anti-c-myc antibodies in culture of native splenocytes, suggesting their highest therapeutic potential as targeted B cell killing agents.

## Introduction

Findings of the last two decades suggest that B cells play a significant role in autoimmune diseases [1], [2]. It may include several pathways, such as production of antibodies (Abs) towards self-antigens, immune complex formation [3], or cytokine and chemokine secretion by autoreactive B cells [4], [5], [6]. It was shown that Abs may be used as markers of the development [7], [8] and progression of autoimmune diseases [9]. Autoreactive B cells may act as antigen-presenting cells in autoimmune diabetes and multiple sclerosis [10], additionally being one of the major cytokine producers in autoimmune disorders [11]. B cells producing Abs to self-antigens act as pathogenic entities, but recent findings indicate that distinct B cell subsets may also have regulatory functions by suppressing unwanted autoaggressive T cells response, and thus, ameliorate the progression of autoimmune diseases [12], [13], [14]. Thus, similar to the T cells [15], B cells may be considered as an important target for the immune intervention.

Basically three B cell-directed strategies exist for treatment of autoimmune disorders: (i) the administration of depleting Abs targeting B cell-specific surface molecules; (ii) targeting B cell receptor (BCR) signaling, which leads to apoptosis of B cells; (iii) inactivation of B cells survival and activation factors. The first approach was developed by several research groups [5], [16], [17], using Abs raised against the B cell surface molecules CD19, CD20, CD21, CD22 and CD23. These Abs have been successfully used for *in vivo* B-cell-directed therapy. However, it should be emphasized that only a limited number of them have been approved for the clinical trials: Rituximab® (human/murine chimeric anti-human CD20(hCD20) monoclonal antibody) [4], Ofatumumab® (human anti-CD20 monoclonal antibody) [18] and Ocrelizumab® (humanized anti-CD20 monoclonal antibody) [19]. The second strategy is focused on Abs specific to BCR-associated transmembrane signaling proteins CD79a and CD79b, almost exclusively exposed on the B cell surface [20]. Inhibition of B-cell survival and proliferation by the blockade of CD40-CD40L interaction [21], [22] may be considered as an example of third approach. Two members of TNF family BAFF (B-cell activating factor) and APRIL (a proliferation including ligand) can also be used as a target for antibody-mediated interruption of BAFF/APRIL signaling pathway [23], [24]. Administration of Belimumab®, a humanized monoclonal antibody against soluble BAFF, has been shown to be beneficial in clinical trials with patients with moderate to severe rheumatoid arthritis [25]. Moreover, some authors reported about administration of intravenous immunoglobulins (IVIG) in therapy of murine experimental rheumatoid arthritis model, and comparison of therapy by IVIG and sialylated IgG Fc molecules (derived from either intravenous immune globulin or human recombinant IgG1). However, mechanism of action of recombinant sialylated IgG Fc is still not known [26].

Nevertheless, obtained drugs have massive side effects and generally are non-specific. A number of patients with systemic lupus erythematosus died in the context of being treated with Rituximab® according to the FDA official alert (www.fda.gov/Drugs/DrugSafety/PostmarketDrugSafetyInformationforPatientsandProviders/ucm126519.htm). Moreover, CD20 antibody-mediated B-cell depletion before EAE induction substantially exacerbated disease symptoms and increased infiltration of encephalitogenic T cells into the CNS. Increased symptom severity resulted from the depletion of a rare IL-10-producing CD1d^hi^CD5^+^ regulatory B-cells subset (B10 cells), since the adoptive transfer of splenic B10 cells before EAE induction normalized EAE in B-cells-depleted mice [12]. Rituximab® treatment has been reported to cause the following serious adverse events such as cardiac arrest, tumor lysis syndrome causing acute renal failure, hepatitis B reactivation and other viral infections, progressive multifocal leukoencephalopathy (PML), immune toxicity with depletion of B cells from 70% to 80% in lymphoma patients, or pulmonary toxicity [27], [28].

Thus, the best immunotherapy should inhibit the pathogenic function without influence on the regulatory abilities of B-cells. The selective elimination of autoreactive B-cells by targeted molecules seems to be the optimal way to realize this approach. We have designed a panel of immunotoxins based either on (i) barnase – ribonuclease from *Bacillus amyloliquefaciens* [29], (ii) catalytic domain of *Pseudomonas* toxin, (iii) catalytic domain of Shiga-like toxin *E.coli*, and (iv) Fc fragment of IgGγ1 antibody. The legibility and toxic properties of the different constructs were thus investigated which of them could represent the best strategy for specific B cells depletion.

## Methods

### Cells

Chinese hamster ovary cells (CHO), C-MYC and 1B4F4 hybridoma cells were cultured in DMEM (GIBCO Invitrogen, USA) with 10% FBS (GIBCO Invitrogen, USA) supplemented with 1x Antibiotic-Antimycotic solution (GIBCO Invitrogen, USA), glutamine (GIBCO Invitrogen, USA), and non-essential amino acids (Gibco Invitrogen, USA).

### Construction of vectors and expression of proteins

The human IgGγ1 Fc domain expression vector (pBudCE4.1/CMV/Lead H/gamma) was obtained previously in our laboratory [30]. The DNA encoding c-myc peptide (EQKLISEEDL) was generated by PCR, sequenced and inserted into the pBudCE4.1/CMV/Lead H/gamma plasmid. Stable CHO cells (CHO-K1 cells, ATCC CCL61) were transfected with pBudCE4.1/CMV/Lead H/c-myc/linker/gamma plasmid by Lipofectanine™ reagent (Invitrogen, USA) and cultured in the presence of Zeocin (0.7 mg/ml) (Invitrogen, USA) in DMEM. Soluble Fc-c-myc protein was purified from culture supernatants of stable CHO cells on Protein G sepharose (GE Healthcare, USA), according to the manufacturer's protocol. The DNA encoding c-myc peptide was generated by PCR, sequenced and inserted into the previously obtained pET22N vector [31]. cDNA encoding the Shiga-like toxin A-subunit was amplified by PCR from *E.coli* (*E. coli* O157:H7) genomic DNA and cloned into the pET22N. To obtain chimeric construct of the ETA-c-myc, the sequence encoding truncated form of ETA (ETA252–608) was amplified by PCR from the plasmid pIG6-4D5MOCB-ETAH6KDEL [32] and fused with a DNA fragment encoding c-myc peptide, generated by PCR. The resulting ETA-c-myc construct was cloned into the pET-22b(+) vector (Novagen, UK). To obtain chimeric construct of His-barnase-c-myc, the DNA fragment encoding c-myc-peptide was reconstituted using a pair of primers, and a flexible peptide linker (Gly_4_Ser)_3_ was added between barnase module and c-myc-peptide. The DNA fragment encoding barnase was amplified from the pSD-4D5scFv-barnase vector [29], subsequently fused with c-myc-(Gly_4_Ser)_3_ fragment and further cloned into the pSD vector. All DNA constructs were verified by sequencing. To produce the recombinant proteins, *E.coli* BL21(DE3) strain was electroporated with pSD-His-barnase-c-myc (or pET22-c-myc-ETA-His) plasmid and incubated in LB medium at 25°C. At OD_550_ = 0.6 to the bacterial suspension was added IPTG to the final concentration of 1 mM. Cell culture was further incubated at 25°C for 12 h. Purification of recombinant proteins was carried out as described earlier [29]. To produce the recombinant protein SLT-c-myc, *E. coli* BL21(DE3) strain was electroporated with pET22N-SLT-c-myc-His plasmid and further incubated in LB medium at 37°C. At OD_550_ = 0.6 to the bacterial suspension was added IPTG to the final concentration of 1 mM. Cell culture was further incubated at 37°C for 6 h, SLT-c-myc protein was expressed in insoluble inclusion bodies. Cells were lysed by sonication and inclusion bodies were washed 3 times by buffer containing 50 mM Tris, 100 mM NaCl, 5 mM EDTA, and 0.5% Triton-X100, pH 8.0. Purified inclusion bodies were resuspended in 8 M urea (∼5 ml per liter of culture), protein solution was loaded on IMAC column (GE Healthcare, USA) in PBS - 8 M urea buffer with subsequent refolding on column using linear gradient of PBS buffer. All prepared proteins were additionally purified on the Superdex 75 column (GE Healthcare, USA). SDS-PAGE was performed in 12% polyacrylamide gel according to the standard protocol. Western blot hybridization analysis was carried out using Hybond-C transfer membrane (Amersham Bioscience, UK) according to the manufacturer's recommendations, using goat anti-c-myc and anti-mouse HRP-conjugated Abs (Sigma, USA). Mouse anti-Fc HRP-conjugated antibody (Sigma, USA) was used in the case of Fc containing molecules. The immunoblots were visualized using ECL Plus Western Blotting Detection System (Amersham, UK) and Bio-Rad VersaDoc 4000 MP imager. Trx-c-myc preparation was describe previously [9].

### In vitro translation in Krebs-2 cells S30 extract

S30 extracts from mice Krebs-2 ascites cells were prepared as described earlier [33]. Translation experiments were performed in a total volume of 10 µl containing 3.5 µl of the S30 extract, 1x Translation Buffer (20 mM Hepes–KOH pH 7.6, 1 mM DTT, 0.5 mM spermidine–HCl, 0.6 mM Mg(CH_3_COO)_2_, 8 mM creatine phosphate, 1 mM ATP, 0.2 mM GTP, 120 mM CH_3_COOK and 25 µM of each amino acid), 2 units of RiboLock Ribonuclease Inhibitor (Fermentas, Latvia), and 2 µl of PBS buffer containing various concentrations of barnase, barnase-c-myc, RNAse A, SLT-c-myc, ETA-c-myc or Trx-c-myc. After 5 min of preincubation at 30°C, 0.25 pmol of m^7^G-capped mRNA encoding firefly luciferase (Fluc) was added [34]. The translation mixtures were incubated at 30°C for additional 45 min, and the luciferase activity was measured using the Dual Luciferase Assay kit (Promega, USA).

### RNAse activity test

Fifty ng of barnase-c-myc, barnase and pancreatic RNAse A were added to 6 µg of total yeast RNA in 10 µl of PBS buffer. After 1 hour incubation at 37°C, the reaction was stopped by incubation at 65°C for additional 30 min with subsequent incubation on ice. Prepared samples were loaded on 1% agarose gel and visualized by ethidium bromide (0.5 µg/ml).

### Binding of C1q complement system component to Fc and Fc-c-myc molecules

96 well plates were coated overnight at 4°C by monoclonal human IgGγ1, Fc domain of monoclonal human IgGγ1 purified after papain hydrolysis, Fc-linker and Fc-linker-c-myc molecules (all proteins in concentration of 10 µg/ml in carbonate buffer pH 9.5). Bovine serum albumin was used as a negative control. C1q component (prepared as described in [35]) in different concentrations was added to prewashed wells in veronal buffer pH 7.4 (VBS++). After 1 hour of incubation wells were washed and treated with rabbit HRP-conjugated anti-C1q Abs. Finally, OD_450_ was measured after incubation with TMB and termination of the reaction by 15% phosphorous acid.

### Incubation of C-MYC and control hybridomas with recombinant ITs

Control and C-MYC hybridomas were resuspended and counted in Goryaev chamber. Cells (10^6^ cells/ml) were added to 24-well plate (Nunc) in 0.5 ml of culture medium. ITs at different concentrations were added to prepared cells. One percent of human serum was added to the cells in the case of Fc-c-myc and Fc. After 72 hours of incubation at 37°C in the presence of 5% CO_2_, the number of live cells in each well was measured using MultiTox-Fluor Multiplex Cytotoxicity Assay (Promega, USA).

### Obtaining of BSA-FITC-c-myc molecule

BSA-FITC molecule was prepared according to the manufacturer's instructions for FITC conjugation (Sigma, USA). The 3 ml of BSA-FITC molecule (1.5 mg/ml) in sodium phosphate buffer pH 7.4 was incubated for 40 min at 20°C with 100 µl of 10 mg/ml MBS (3-Maleimidobenzoic acid N-hydroxysuccinimide ester, Sigma, USA) resuspensed in DMSO. Further sample was desalted on HiTrap Desalt (GE Healthcare, USA) column to sodium phosphate buffer pH 6.0. Eluted BSA-FITC-MBS molecule was added to 500 µl of 10 mg/ml solution of c-myc peptide with N-terminal cystein (C**EQKLISEEDL**, synthesized by solid-state methodology in IBCh RAS). After 3 hours incubation at 20°C sample was dialyzed against PBS. To check the ability of obtained BSA-FITC-c-myc molecule to bind anti-c-myc monoclonal Ab (mAb), 50 µg of conjugate were incubated with 100 µg of anti-c-myc mAb for 1 h at 37°C. After incubation sample was loaded on Superdex 200 column (GE Healthcare, USA). Control injections of non-mixed BSA-FITC-c-myc and anti-c-myc mAb were performed. Fractions from GFC were subjected on PAGE and visualized by coomassie staining and measurement of FITC fluorescence emission.

### Immunization of BALB/c mice with KLH-c-myc molecule

The 600 µl of KLH molecule (6 mg/ml) (Fluka) in sodium phosphate buffer pH 7.4 were incubated for 40 minutes at 20°C with 50 µl of 10 mg/ml MBS resuspensed in DMSO (Sigma, USA). Further sample was desalted on HiTrap Desalt (GE Healthcare, USA) column to sodium phosphate buffer pH 6.0. Eluted conjugate was incubated with 300 µl of 10 mg/ml solution of c-myc peptide with N-terminal cystein. After 3 hours incubation at 20°C sample was dialyzed against PBS. BALB/c mice were immunized s.c. by 75 µg of KLH-c-myc in complete Freund's adjuvant. After 21 days, mice were boosted by i.p. injection of 75 µg KLH-c-myc in PBS.

### Incubation of isolated splenocytes with recombinant ITs

Spleens of immunized or intact BALB/c mice were isolated on 24 day after first immunization. Isolated splenocytes were resuspended and further counted in Goryaev chamber. Cells (3×10^6^ cells/ml) were added to 24-well plate (Nunc) in 1 ml of culture medium. ITs were added to prepared cells in the following concentrations: barnase and barnase-c-myc (0.55 µM), Fc-linker and Fc-linker-c-myc molecules (0.1 µM). One percent of human serum was added to the cells in the case of Fc-c-myc and Fc analysis. Trx-c-myc (4.5 µM) molecule and 1% human serum were used as a negative control. Samples of splenocytes were analyzed in terms of anti-c-myc Abs production and percentage of c-myc-specific B cells every 24 hours.

### Analysis of antibody production by isolated splenocytes

>Samples of cell supernatants were analyzed by ELISA on 24, 48 and 72 hours after administration of ITs to splenocytes. 96 well plates were coated by goat anti-whole anti-mouse Ab (Sigma, USA) or by Trx-c-myc recombinant molecule (0.25 µg/well of protein in carbonate buffer pH 9.5) and incubated overnight at 4°C. Cell supernatants in different dilutions were added to prewashed wells and incubated for 1 hour at 37°C. Further wells were washed and incubated with goat anti-mouse Fc HRP-conjugated Ab (Sigma, USA) for 1 hour at 37°C. Finally, OD_450_ was measured after incubation with TMB and termination of the reaction by 15% phosphorous acid.

### Flow cytometry

One million of splenocytes were centrifuged for 10 min at 400 g. Precipitated cells were washed by PBS twice. BFC solution and anti-B220-APC Ab (eBioscience, USA) in PBS-1%BSA were added to cells and further incubated for 40 minutes at 4°C. Stained cells were centrifuged for 10 min at 400 g, resuspended in FACS buffer (0.1% BSA, 0.02% Azide, 50 ug/ml propidium iodide in PBS) and analyzed on flow cytometer FACSdiva (Becton Dickinson, USA).

## Results

### Construction and purification of recombinant IT

**Figure 1:**
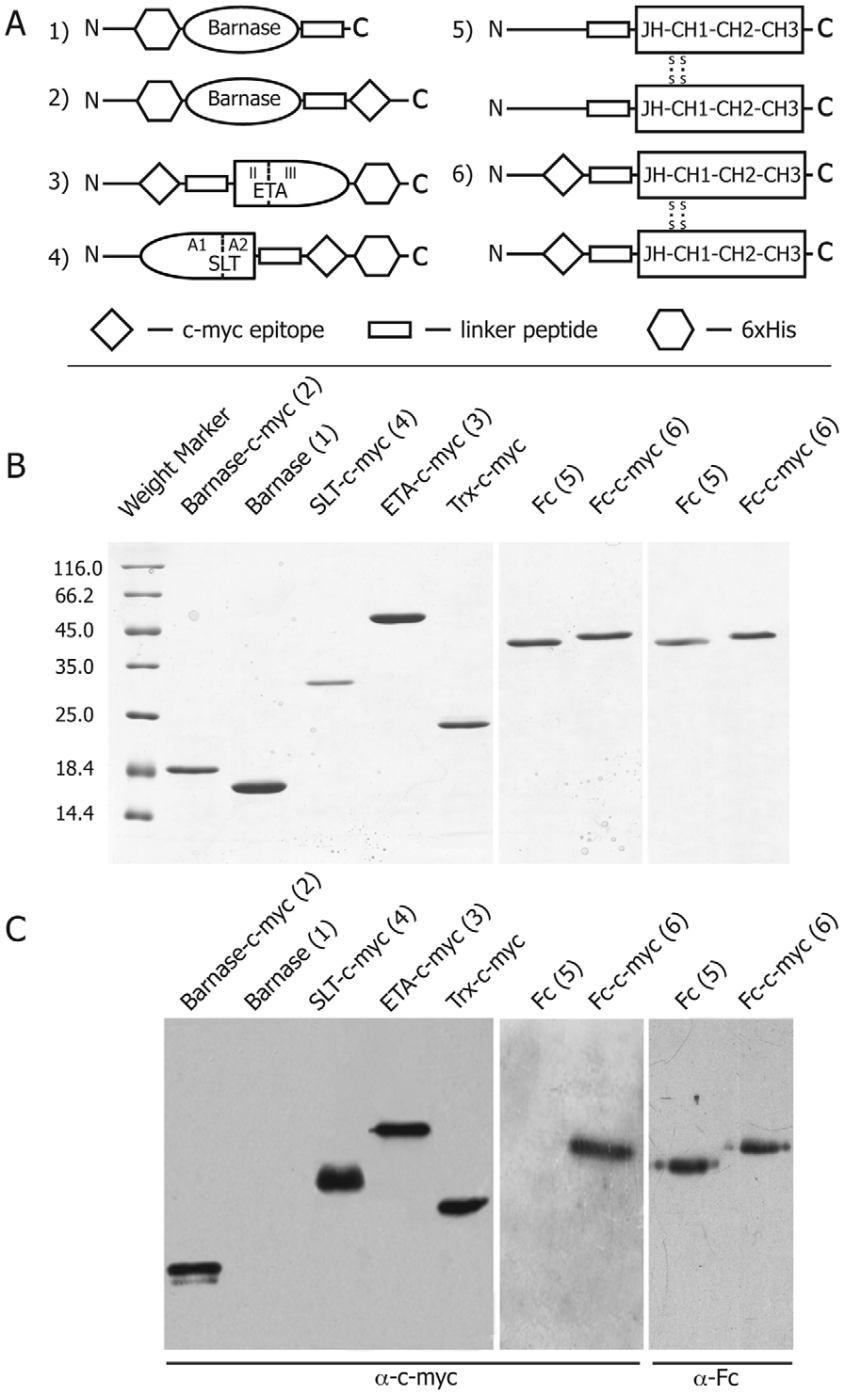
Construction and purification of recombinant IT. (A) Schematic representation of the designed ITs, fused with c-myc epitope sequence, based on barnase (1 and 2), catalytic (III) and translocation (II) domains of *Pseudomonas* toxin ETA (3), catalytic (A1) and binding (A2) domains of Shiga-like toxin *E.coli* (4) and Fc domain of human antibody IgGγ1 (5 and 6). (B) The purity of prepared recombinant molecules was confirmed by 12% SDS-PAGE. (C) The presence of the c-myc epitope was shown by hybridization of recombinant ITs with anti-c-myc mAbs, produced by selected hybridoma.

We designed and obtained a set of IT of various cytotoxicity based on barnase (Figure 1A 1,2), *Pseudomonas* toxin (Figure 1A 3), Shiga-like toxin (Figure 1A 4) and human Fc domain (Figure 1A 5,6). Recombinant molecules (2, 3, 4, 6) were fused with c-myc epitope. We also designed control molecules, which did not contain c-myc epitope (1, 5). All recombinant molecules barnase, barnase-c-myc, ETA-c-myc, SLT-c-myc contained 6xHis tag. Proteins were purified using affinity chromatography and additionally subjected to GFC on Superdex75/200 columns depending on the protein size. Purity of the obtained IT was tested by denaturating PAGE (Figure 1B) and was more than 95%. Purified IT 2, 3, 4 and 6 were recognized by mAb towards c-myc epitope produced by selected hybridoma (Figure 1C), while control molecules 1 and 5 showed no hybridization with these immunoglobulins.

### Functional activity and specificity of the obtained IT in vitro

**Figure 2:**
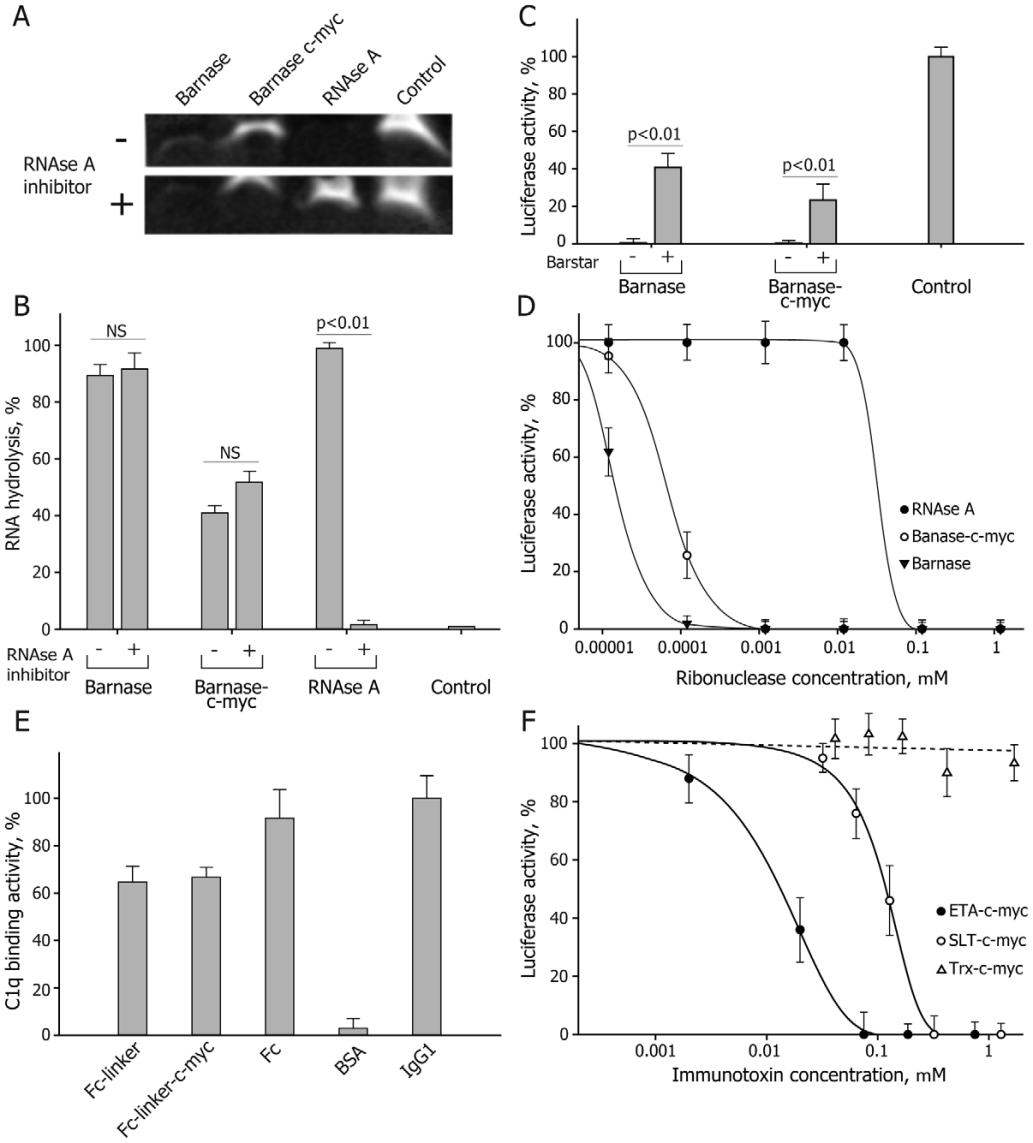
Functional in vitro activity of obtained IT. (A) Representative hydrolysis of yeast RNA by recombinant barnase, barnase-c-myc and RNAse A in the absence or presence of RNAse inhibitor. (B) Quantitative analysis demonstrates that presence of pancreatic inhibitor of RNAse A does not affect the activity of barnase and barnase-c-myc. (C) Suppression of luciferase RNA translation in S30 ascites cell extract by barnase and barnase-c-myc in the presence or absence of barstar. (D) Barnase and barnase-c-myc are still inhibit luciferase RNA translation in concentrations of three orders of magnitude less than those for RNAse A. (E) Binding of the C1q component of the complement system to the Fc and Fc-c-myc molecules. Full-size Abs and their Fc fragments were used as a positive control. Recombinant Fc and Fc-c-myc molecules demonstrate the same affinity compared with native immunoglobulins. (F) Suppression of luciferase RNA translation in S30 ascites cell extract by *Pseudomonas* and SLT toxins. Activity of the RIPs was determined by decreasing of the level of luciferase mRNA translation in mouse Krebs-2 ascites cells S30 extracts depending on the IT concentration. Total inhibition of protein synthesis was detected at the ETA-c-myc concentration of 75 nM which was five times lower than for the SLT-c-myc molecule (320 nM). Bars in all experiments represent standard deviation (n = 3).

To estimate the cytotoxic potential of the obtained IT, we analyzed their functional activity and specificity *in vitro*. In the case of barnase and barnase-c-myc we tested RNAse activity by incubating these proteins with yeast RNA in the presence and absence of pancreatic RNAse inhibitor to detect possible contamination of samples with co-purified prokaryotic RNAses (Figure 2A). Quantitative analysis of RNA hydrolysis in the absence of RNAse inhibitor performed by TotalLab software showed that barnase hydrolyzed yeast RNA with the same rate when compared with RNAse A, while activity of barnase-c-myc was twice lower than that of barnase and RNAse A (Figure 2B). We found that the pancreatic RNAse inhibitor did not influence on the activity of barnase and barnase-c-myc, suggesting that observed RNAse activity completely belonged to barnase and barnase-c-myc molecules. Barnase activity is not inhibited by intracellular RNAse inhibitors [36] and is affected only by a specific inhibitor – barstar . To clarify this, we performed *in vitro* translation experiments in S30 cytoplasmic extract of Krebs-2 ascites cells (Figure 2C,D). The extract was pre-incubated with increasing amounts of barnase, barnase-c-myc and RNAse A, and then tested for translation of the luciferase mRNA. We showed that barnase and barnase-c-myc were still active in concentrations of three orders of magnitude less than those for RNAse A (Figure 2D). Taking into the account that RNAse A is even more active than barnase as an enzyme [37], [38], this observation suggests that barnase-mediated RNA hydrolysis is substantially non-affected by intracellular RNA inhibitors.

C1q-binding activity of the Fc and Fc-c-myc molecules was determined by ELISA (Figure 2E). Briefly, immobilized Fc and Fc-c-myc molecules were incubated with C1q component of the complement system with further hybridization with anti-C1q Abs. Native IgGγ1 Abs and Fc domains of these Abs were used as a positive control. We showed that recombinant Fc and Fc-c-myc molecules interacted with C1q component of the complement system with a similar affinity as compared with native IgGγ1 Abs.

ETA and SLT toxins affect components of eukaryotic translational machinery. To test their activities, we used *in vitro* translation system based on S30 cytoplasmic extract of Krebs-2 ascites cells. The extract was pre-incubated with increasing amounts of ETA-c-myc and SLT-c-myc and then assayed for translation of the luciferase mRNA. Recombinant thioredoxin fused with c-myc epitope (Trx-c-myc) purified in the same conditions was used as negative control. Figure 2F indicates the decrease in translation efficiency in the presence of tested proteins, with complete inhibition of protein synthesis at the ETA-c-myc concentration of 75 nM, which was five times lower than that for the SLT-c-myc molecule (320 nM). Trx-c-myc didn't have any detectable effect on eukaryotic translational machinery being used in concentrations up to 1800 nM. These data indicate that both ETA-c-myc and SLT-c-myc ITs possess properties of highly effective inhibitors of the protein synthesis, at least *in vitro*.

### Functional activity and specificity of the obtained ITs ex vivo

**Figure 3:**
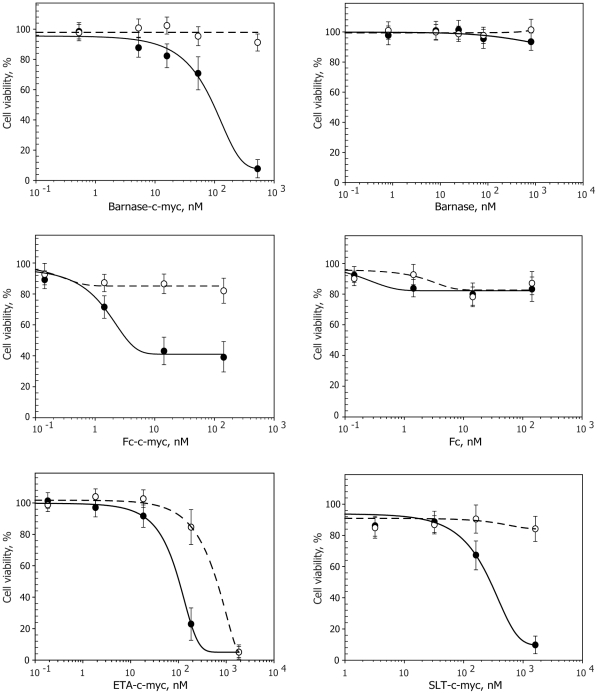
Functional activity and specificity of the obtained immunotoxins tested ex vivo. ITs' activity was determined by the *ex vivo* elimination of the targeted C-MYC (solid line) and irrelevant (dotted line) hybridoma cell lines depending on the IT' concentration. IT containing c-myc sequence selectively killed targeted hybridoma, keeping the irrelevant cells alive. Barnase-c-myc and Fc-c-myc showed rather high cytotoxicity and specificity. ETA-c-myc was highly cytotoxic for both cell lines. SLT-c-myc molecule showed intermediate level of the cytotoxicity and specificity. Bars represent standard deviation (n = 3).

To determine activity of the obtained ITs *ex vivo,* purified recombinant proteins were incubated with C-MYC hybridoma cell line; besides, we used 1B4F4 hybridoma producing irrelevant Abs as a control. Incubation of the ITs with cells was performed in 24-well plates for 72 h. ITs were added at concentrations ranging from 0.1 to 2000 nM in order to obtain correct calculations of the IC_50_ values. The number of alive cells was measured after 72-h incubation. Obtained data were used for plotting the curves showing the dependence of the alive cells amount as a function of ITs concentration (Figure 3). Recombinant molecules that did not contain c-myc sequence were used as a negative control. In the case of Fc-c-myc and Fc molecules, 1% human serum was used as a source of components of the complement system for effective elimination of the targeted cells via CDC mechanism.

We detected high functional activity and specificity of the obtained recombinant molecules. Based on the experimental data we calculated LD_50_ of the recombinant toxins towards C-MYC hybridoma cell line (Table. 1). Fc and barnase molecules, which did not contain c-myc sequence, served as a negative control in an addition to the control cell line. It is clearly seen that selective elimination of the C-MYC hybridoma cell line took place only in case of application of the IT with c-myc sequence. ETA-c-myc molecule showed high cytotoxic potential but unexpectedly for both cell lines. IT based on SLT showed medium values of the cytotoxicity and specificity as compared to the other ITs. The most promising results were obtained for the IT based on the barnase and Fc molecules, which showed the best cytotoxicity/specificity ratio (Table. 1).

**Table 1:** Immunotoxin LD_50_ determined on model C-MYC and control 1B4F4 cells.

Immunotoxin	c-myc epitope	LD_50_ C-MYC cells, nM	LD_50_ 1B4F4 cells, nM	Specificity**
*Pseudomonas* toxin (ETA)	+	5·10^1^	10^3^	20
Shiga-like toxin (SLT)	+	4.4·10^2^	10^5^ *	230*
Barnase	+	1.1·10^1^	10^5^ *	900*
	−	10^4^ *	10^5^ *	-
FcIgGγ1 (Fc)	+	2.0	10^4^ *	5000*
	−	10^4^ *	10^5^ *	-

*More than this value

**LD_50_(1B4F4)/LD_50_(C-MYC)

**Figure 4:**
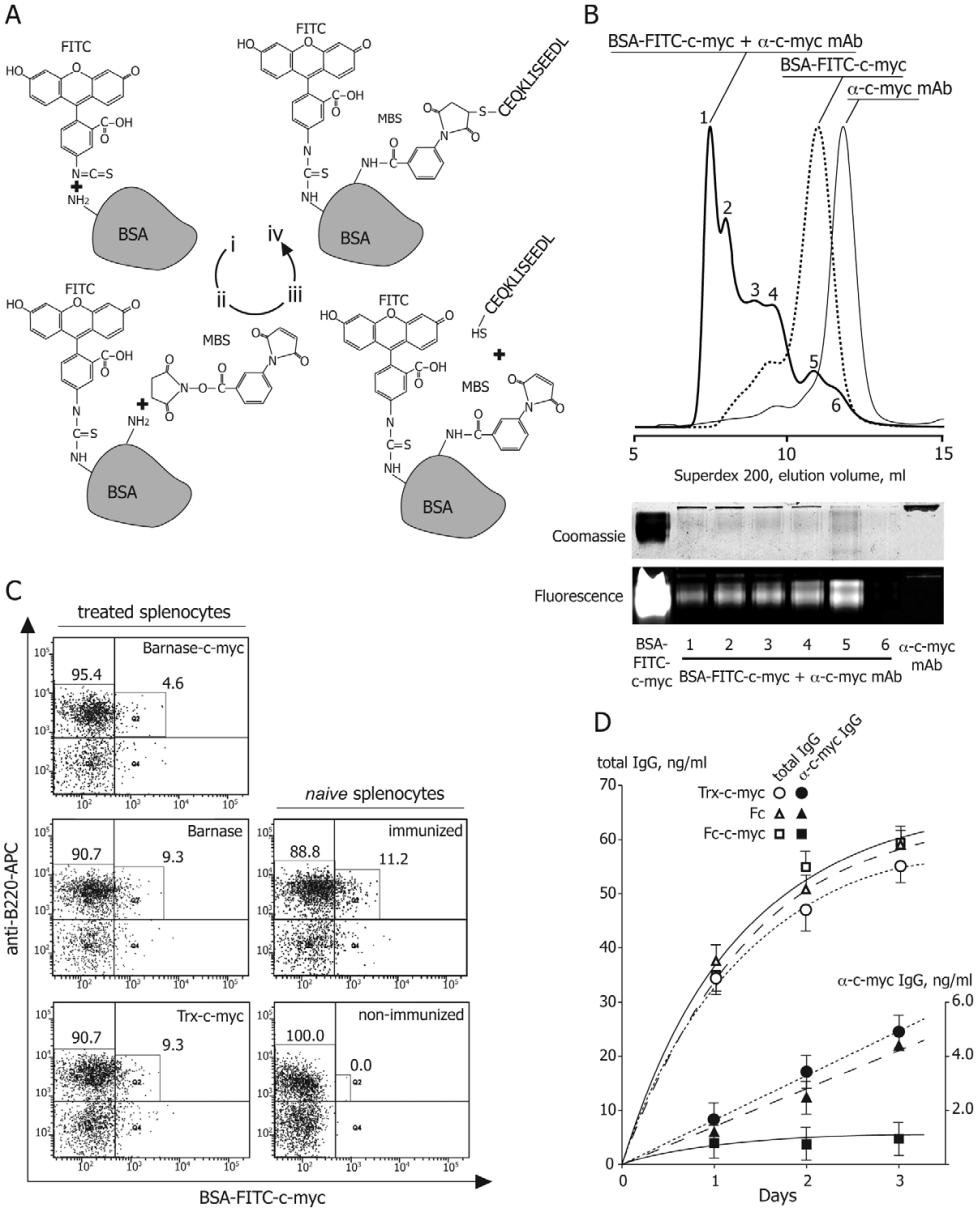
Barnase-c-myc and Fc-c-myc specifically deplete anti-c-myc B cells and decrease production of anti-c-myc Ab in culture of splenocytes, isolated from mice immunized by KLH-c-myc. (A) Procedure of obtaining of BSA-c-myc conjugate labeled by FITC for flow cytometry detection of targeted anti-c-myc B cells. (i) Conjugation of BSA with FITC using coupling of isothiocyanate with primary amino groups. (ii) Reaction of free primary amino groups of BSA-FITC with MBS. (iii) Coupling of second functional group of MBS with SH group of N-terminal cysteine, flanking c-myc peptide. (iv) Final bifunctional reagent BSA-FITC-c-myc, which is capable to react with BCR on the surface of B cells due to the attached c-myc peptide and carrying fluorescein group for detection. (B) Binding of anti-c-myc mAb to BSA-FITC-c-myc in solution. Superdex 200 gel-filtration profile of BSA-FITC-c-myc alone (dashed line) and preincubated with anti-c-myc mAb (solid line). Anti-c-myc mAb are shown by thin solid line. Fractions from gel-filtration chromatography of BSA-FITC-c-myc preincubated with anti-c-myc mAb were subjected on PAGE and visualized by coomassie staining and measurement of FITC fluorescence emission (bottom panel). (C) Representative flow cytometry analysis of native splenocytes, isolated from non-immunized and immunized by KLH-c-myc mice, using BSA-FITC-c-myc and anti-B220 APC-labeled antibody (right panels). Approximately 11% of all B cells carry BCR specific for c-myc peptide (B220^high^ BSA-FITC-c-myc^high^, Q3 quadrant). Representative flow cytometry analysis of splenocytes, isolated from mice immunized by KLH-c-myc, treated by barnase-c-myc, barnase and Trx-c-myc (left panels from top to bottom). (D) Analysis of total antibody titer (open figures) and anti-c-myc Abs (filled figures) in samples of splenocytes, isolated from KLH-c-myc immunized mice, treated by Trx-c-myc (circles), Fc (triangles) and Fc-c-myc (squares) molecules. Bars represent standard deviation (n = 3).

To test barnase- and Fc-based IT on native B cells, BALB/c mice were immunized with KLH-c-myc to raise B cells producing anti-c-myc Abs. We obtained BSA-FITC-c-myc conjugate (Figure 4A) to uniquely determine c-myc-specific B cells among total B cells pool. This double labeled protein was tested in terms of anti-c-myc mAb binding by migration of peak of anti-c-myc mAb complexed with BSA-FITC-c-myc conjugate (Figure 4B). Flow cytometry analysis revealed that splenocytes, isolated from mice immunized with KLH-c-myc, contained 11.2% of B cells carrying anti-c-myc BCR (Figure 4C, right panels). Splenocytes from immunized mice were treated by barnase- and Fc-based IT in optimal concentrations in order to test their cytotoxicity and specificity. In case of barnase-c-myc we succeeded to show twice decreasing of the c-myc-specific B cells, while other B cells were alive (Figure 4C, left panels). Administration of Fc and Fc-c-myc molecules resulted in unspecific staining of cells during the flow cytometry analysis. In order to analyze their effect, we performed comparable analysis of total and anti-c-myc Abs titer, produced by splenocytes treated by Fc, Fc-c-myc and Trx-c-myc. We detected 5-times decreasing of concentration of anti-c-myc Ab in splenocytes samples incubated with Fc-c-myc molecule, compared to control samples (Figure 4D). Importantly, that we didn't observe any significant changes in concentration of total IgG suggesting high selectivity of Fc-c-myc molecule, specifically decreasing production of anti-c-myc Ab by B cells.

## Discussion

**Figure 5:**
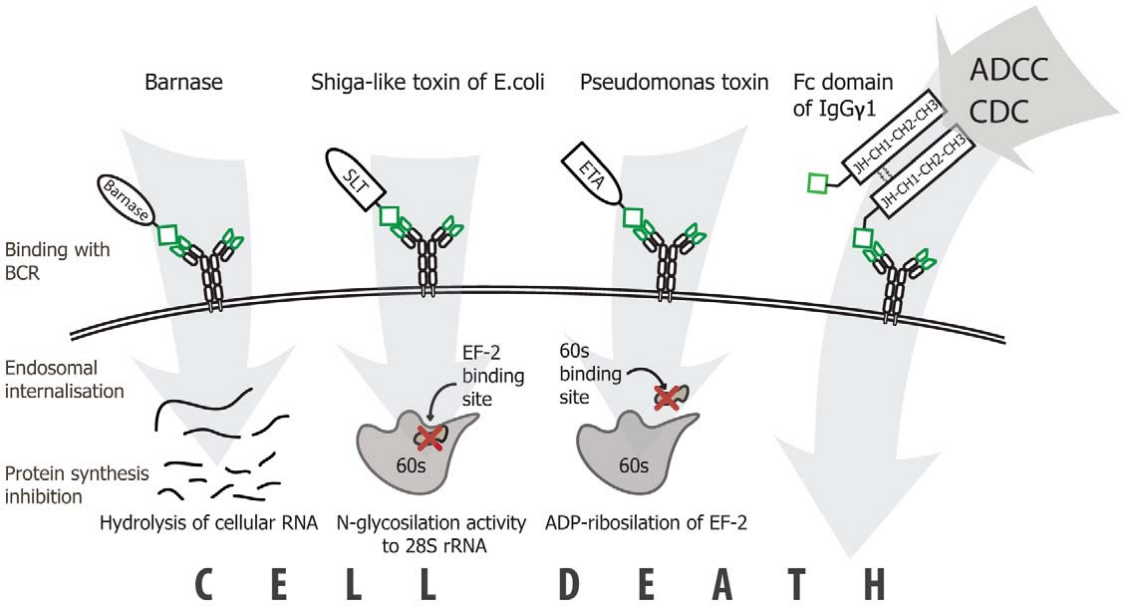
The mechanism of action of designed ITs. Barnase, Shiga-like toxin (SLT) and *Pseudomonas* toxin (ETA) are recognized by BCR due to the attached epitope with subsequent endosomal internalization followed by inhibition of protein synthesis via hydrolysis of cellular RNA, N-glycosylation of 28S rRNA or ADP-ribosylation of EF-2 respectively. In the case of Fc domain, targeted cell death occurs via antibody dependent cellular cytotoxicity (ADCC) or complement dependent cytotoxicity (CDC).

Autoreactive B cells are one of the key players in many autoimmune pathologies. These aggressive pathogenic cells cause severe tissue damage and malfunction through several pathways. Antibody-mediated autoimmune diseases may be ameliorated by immunosuppressive drugs, corticosteroids or plasmapheresis, which all act only transiently or may be reason of general immunodeficiency. Elimination of pathogenic B cells by different ITs such as MOG-Fc, MOG-ETA and MBP-ETA was reported previously [39], [40], [41]. However, there is still no effective therapy based on the depletion of disease-associated B cells in autoimmune patients. Here we succeeded to perform comparative investigation of ITs' set, based on different molecules and mechanisms of action. We have designed a panel of ITs based on (i) barnase – ribonuclease from *Bacillus amyloliquefaciens*, (ii) catalytic and translocation domains of *Pseudomonas* toxin, (iii) catalytic domain of Shiga-like toxin *E.coli*, and (iv) Fc fragment of IgGγ1 antibody fused with targeting c-myc sequence. In general, these molecules consist of two parts: an effector domain (toxin, antibody fragment, RNAse) and a delivery domain (part or full length autoantigen) (Figure 5).

The targeting motif of ITs may selectively interact with pathogenic B cells via their BCR and cause a cell death due to the attached or internalized toxin. Barnase and RIP inhibit protein synthesis via hydrolysis of cellular RNA [36], [42] and knocking out of components of eukaryotic translational machinery respectively [43], [44]. A-subunit of Shiga-like toxin consists from two parts: cytotoxic part A1 and binding part A2 (Figure 1A). During intracellular toxin transport the furin site between A1 and A2 is cleaved by furin resulting in the separated carboxy-terminal A2 fragment and cytotoxic A1 fragment (27 kDa), the last one subsequently escapes from the early endosome [45]. In our study all artificial elements, including linker, c-myc and 6xHis tags are fused with A2 fragment. Thus, after endocytosis and furin-depended release of fully natural fragment A1, it will function independently according preassigned cell-killing program. Seemingly, nucleotide sequence of designed ETA-c-myc molecule includes translocation domain II of *Pseudomonas toxin,* fused with c-myc sequence (Figure 1A), and released in endosome natural cytoxic domain III, which further independently delivered to the cytosole [44]. Thus, chimerical bacterial RIPs described in this study have natural ability to migrate from endosome to the cytosole and kill targeted cell. Barnase hasn't such natural properties. However, it was reported previously, that barnase was released from endosomes to cytosole [36]. We showed that, in contrast with activity of RNAse A, inhibitors existing in eukaryotic cell extracts did not affect barnase-mediated RNA degradation. Our data are in accordance with previously findings [36], thus, the designed ITs based on Barnase should not be affected by eukaryotic RNAse inhibitors after their internalization. In the case of Fc-domain-containing molecules, depletion of pathogenic B cells occurs via the ADCC (antibody dependent cellular cytotoxicity), CDC (complement dependent cytotoxicity) mechanisms and probably by recently discovered ozone formation [46]. We showed the IT functional activity, e.g. blocking of RNA translation and subsequently ribosomal protein synthesis by ETA and SLT molecules, binding of recombinant Fc fragment to C1q component of the complement system, and finally, the ribonuclease activity of barnase. These experiments suggest that all ITs may potentially kill cells after simple binding on the surface or subsequent internalization, if required. The designed ITs were recognized by mAb produced by targeted hybridoma as shown by the Western-blotting analysis. Thus, they may bind specifically to respective BCR on the cell surface and do not interact with irrelevant cells. Further we compared their legibility and toxic properties to estimate which of these three approaches would be the best choice for specific B-cells depletion. With this purpose we calculated the LD_50_ of the recombinant toxins for C-MYC and control hybridoma cell lines. We have shown that selective elimination of the C-MYC hybridoma cells took place only in case of application of the IT with c-myc sequence, while Fc and barnase molecules without c-myc sequence were inactive. ETA-c-myc molecule showed high cytotoxic effect for both target and control cell lines. IT based on SLT showed intermediate cytotoxicity and specificity as compared with other immunotoxins. Importantly, that *ex vivo* data of LD_50_ were correlated with IC_50_ values, obtained from *in vivo* experiments, suggesting that cytotoxic domains of RIPs internalize into the cytosole of targeted cells and this process is occurred with same efficiency for both SLT and ETA. The most promising results were obtained for the IT based on the barnase and Fc molecules, which showed the best cytotoxicity/specificity ratio (Table. 1), approximately 900 and 5000 units respectively. According to flow cytometry data and IgG antibody titer we showed that these ITs were able to specifically deplete anti-c-myc B cells and decrease production of ant-c-myc Ab in culture of splenocytes, isolated from mice immunized by KLH-c-myc.

In summary, we present that SLT and ETA molecules within their high toxicity also revealed increased unspecific cells depletion, which is in accordance with previously published results concerning massive side effects of these ITs. We also report that barnase and Fc domain are the most actual molecules to design killers of pathogenic B cells, as they exhibited the least side toxicity in the reported ITs' set studied. The Fc molecule has additional benefit properties due to its high stability in the blood stream and minimal potential immune response. The presented approach and selected agents can be efficiently used for design of various medications for the directed treatments of autoimmune diseases via selective depletion of the aggressive pathogenic B cells.
